# Preschoolers' prosocial behavior in groups—Testing effects of dominance, popularity, and friendship

**DOI:** 10.3389/fpsyg.2025.1478493

**Published:** 2025-02-11

**Authors:** Anne Katerkamp, Lisa Horn

**Affiliations:** Department of Behavioral and Cognitive Biology, University of Vienna, Vienna, Austria

**Keywords:** prosociality, social relationships, peers, kindergarten, preschool, contextual approach to development, ecological validity

## Abstract

Prosocial, other-benefitting behavior is a fundamental aspect of human social behavior. In the *microsystem* of their natural social groups, preschool children have to decide not only whether, but also whom to share with and help on a daily basis. In a study with 108 preschool children from five different childcare facilities in Vienna, we examined how individual measures of dominance and popularity of both the actor and the recipient, as well as their friendship influenced their prosocial behavior in a group setting. We assessed popularity and friendship with age-appropriate sociometric interviews and tested two types of dominant behavior (i.e., contest and scramble) as well as two types of prosocial behavior (i.e., sharing and helping) in groups of familiar peers. Participants were tested in their regular socio-ecological niches, thereby preserving and taking into account social dynamics that influence—and are influenced by—interactions within the group. We found that both types of prosocial behavior were directed more often toward friends than toward children that were not considered as friends. Likelihood to share was increased by both the actor's and the recipient's dominance in the contest game. Furthermore, we found that helping was preferentially performed by as well as directed toward older children and that dominant children more frequently received help. Group size and testing sequence had some additional effects on sharing and helping. Our findings suggest that sharing and helping have similar as well as different antecedents and underlying motivations and depend on social relationships between the children. Such effects can be examined more effectively when taking a developmental-ecological approach and investigating prosocial behavior in children's natural social environment.

## 1 Introduction

To help and share with others are fundamental aspects of human social behavior. Developmental psychology has long been interested in the early ontogeny of this so-called prosocial behavior (i.e., actions apparently benefiting another agent without providing a direct pay-off for the actor; Paulus, 2018). To date, a considerable number of well-controlled laboratory experiments have probed children's motivations for prosocial actions as well as predictors, mechanisms, and outcomes of prosocial behavior (Davidov et al., 2016). In these experiments, children's prosocial behavior has mostly been studied individually, where one child is required to make prosocial choices based on experimenter narratives, often directed to a hypothetical recipient. Recently, however, there has been a renewed interest in examining behavior not only within the developing individual, but also in its relation to the developing individual's everyday environment (Dahl, 2017). Such a developmental-ecological perspective is based on the premise that human development results from a dynamic interaction between the individual and the individual's environment (Bronfenbrenner and Morris, 2007): on the one hand, the developing organism is influenced by its surroundings (e.g., objects, other individuals), resulting in behavioral adaptations; on the other hand, the environment is shaped through the behavior of the individuals living in it. This is particularly relevant with regard to a developing child's socio-ecological environment, which is shaped by their relationships with others and their position in the affiliative and hierarchical networks of their social group (Horn et al., 2022). To gain a more complete picture of prosocial development, it is therefore crucial to use a developmental-ecological approach and investigate prosocial behavior directly in children's natural social environment and in interactions with familiar social partners.

One of the early proponents of an ecological perspective on children's development was Urie Bronfenbrenner. Bronfenbrenner's seminal bioecological model defines development as both change and stability of biopsychological characteristics of human beings over the life course and across generations (Bronfenbrenner, 1974). The model proposes that development is the result of a complex interplay between proximal processes, person characteristics, context, and time (Bronfenbrenner and Morris, 2007). The nature of a given context has the potential to affect social interactions and developmental processes occurring in this specific context, depending also on the characteristics of the interacting individuals. Bronfenbrenner and Morris (2007) describe the immediate environment of a child, in which direct social interactions take place, as their *microsystem* (e.g., their family). A developing child typically moves within several contextual systems. By the age of three, most children attend preschool and are therefore part of a *microsystem* with peers that differs in several aspects from the family context. Preschool peer groups are usually larger social systems and comprise more varied social partners and relationships than family units (Santos and Vaughn, 2018). Moreover, peers have similar competences and exchanges with peers therefore tend to be more symmetrical and reciprocal than child-adult interactions (Brownell et al., 2006). This suggests that preschool peer groups offer a unique context for studying the development and expression of prosocial behavior. Eisenberg and colleagues heuristic model of prosocial behavior (Eisenberg et al., 2006; Spinrad and Eisenberg, 2023) stresses that the expression of prosocial behavior is influenced not only by individual factors (e.g., genes, gender, affective state), but also by contextual factors, such as situation characteristics or the social environment. Understanding prosocial behavior in children thus requires a multifaceted approach that considers both individual and contextual influences, as well as the biological-ecological underpinnings of these behaviors (Spinrad and Eisenberg, 2023).

Prosocial behavior emerges early in human ontogeny (Silk and House, 2011). Already in the second year of life children help adults in simple tasks (e.g. handing an experimenter an out-of-reach object; Warneken and Tomasello, 2007). Prosocial behavior develops further during the preschool period, increasing in frequency as children get older, develop more sophisticated socio-cognitive skills, and are more strongly influenced by social norms (Martin and Olson, 2015; Silk and House, 2011; Flook et al., 2019). The nature of children's prosocial behavior is multidimensional (Paulus, 2018), with each occurrence of prosocial behavior being the result of a complex interplay between individual and contextual factors (Spinrad and Eisenberg, 2023). Moreover, early prosocial behavior should not be considered as one coherent trait, but different types of prosocial behavior such as sharing and helping likely depend on distinct motivations and mechanisms in children (Paulus, 2018). Sharing is described as voluntarily giving some or all of a resource to another individual (Spinrad and Eisenberg, 2023), whereas helping is described as facilitating another individual to achieve a goal by providing assistance in whatever way needed (Warneken and Tomasello, 2007). Sharing has been argued to depend more strongly on children's moral norms and fairness principles (e.g., Fehr et al., 2008; Schmidt and Sommerville, 2011). Such fairness principles are likely early-emerging, possibly even innate, tendencies that are continuously shaped and enhanced by social experiences throughout an individual's life (Geraci, 2022). Helping, on the other hand, might be based on an inclination to see goals completed or a general motivation to socialize with others (e.g., Carpendale et al., 2014; Michael and Székely, 2019; for a detailed discussion about the potential underlying mechanisms of different types of prosocial behavior see Dunfield, 2014; Paulus, 2018). Around preschool age, children get more selective when acting prosocially. Classic observational studies show that children exhibit a shift toward peer-orientation during this time (Howes et al., 1988), as they become more exposed to an environment where they have the opportunity to socialize with a variety of peers (Howes, 1983; see also Martin et al., 2013). In the complex social environment of a preschool group, children have to decide not only whether to act prosocially or not, but also who among their peers to help or share with. Therefore, it is likely that the expression of prosocial behavior in this environment depends on characteristics of the actor, the recipient or both of them, such as their dominance, popularity, and friendship.

### 1.1 Dominance and prosociality

For children in peer groups it is important to gain resources, such as material objects or alliances with and support from others (Hawley and Bower, 2016). Children's natural differences in their ability to prevail in resource competition result in social dominance and in their position in the group's dominance hierarchy (Hawley, 1999). Dominance has been defined as an individual's ability to gain access to and/or maintain control over resources (Hawley, 2002) and can be influenced by certain person characteristics. Hawley (2002) found that older children were rated as more dominant by their preschool teachers and were more successful at controlling a valuable resource in a dyadic play situation with a peer. Results regarding an influence of gender on dominance, however, are inconclusive. When observing children's interactions among familiar peers, Charlesworth and La Freniere (1983) found that boys exhibited higher dominance by maintaining access to a valuable toy significantly longer than girls. However, other studies suggest that both genders can achieve high dominance, with females often using more subtle strategies to do so (cf. Pellegrini et al., 2007). That dominance plays a significant role in preschooler's peer interactions is supported by the fact that, as early as 3 years of age, children infer others' dominance via social cues such as age or decision power and expect dominant individuals to be more competent in games and have better access to resources (Charafeddine et al., 2015).

Experimental evidence points to a negative association between children's dominance and their prosocial behavior when sharing resources. Preschoolers that prevail in resource conflicts with peers have been found to donate fewer valuable items to an anonymous recipient (Guinote et al., 2015) and to acquire more resources than their partners in cooperative tasks (Grueneisen and Tomasello, 2017). Similarly, preschoolers with higher teacher-rated dominance had a tendency to withhold valuable items from a familiar peer in a resource allocation experiment (Horn et al., 2024b). In natural social groups, however, children sometimes use seemingly prosocial actions to acquire and maintain resources, such as sharing a non-preferred toy with a peer in order to gain access to a preferred toy (Hawley, 1999). In a longitudinal study conducted with children across a whole year of preschool, dominance was positively associated with aggression and coercion at the beginning of the study period, whereas dominant children were particularly prosocial toward the end of the school year (Roseth et al., 2010). When investigating whether the recipient's dominance affected children's prosocial choices, Charafeddine et al. (2016) found that 3-4-year-olds preferentially donated valuable items to a dominant puppet in a forced-choice sharing task, while 5-year-olds did not show a clear preference. Moreover, more than 90% of the tested 8-year-olds gave a bigger piece of chocolate to the subordinate, suggesting that the influence of moral norms and fairness principles that may underlie sharing gets stronger with age (cf. Schmidt and Sommerville, 2011). These findings suggest the association between dominance and prosociality might not be as straightforward as experimental studies suggest and that socio-ecological factors such as the stability of hierarchical structures in a social group might influence whether and how strongly dominance affects prosocial behavior in preschoolers. Such associations can only be tested in the *microsystem* of the peer group, where children interact regularly and over an extended period of time. Further, as is shown in Spinrad and Eisenberg's (2023) heuristic model of prosociality, dominance might not be the only modulating factor of prosocial behavior in natural peer groups.

### 1.2 Popularity and prosociality

Popularity can be defined as a measure of peer acceptance or likeability, with popular individuals having a higher status and a more central position within the group (McDonald and Asher, 2018). Neither age nor gender were found to have a clear influence on popularity in preschool children (Walden et al., 1999). However, observations of peer interactions suggest that popularity and dominance are linked, with higher dominance often going along with more popularity in children (Hawley, 2002; Vaughn and Waters, 1981). Dominant individuals receive more attention from their peers (La Freniere and Charlesworth, 1983), pointing toward their central position in the group. In addition, gender seems to interact with popularity and dominance. Sebanc et al. (2003) found that in same-sex interactions around a valuable resource, more dominant boys were more popular among their peers than less dominant boys, whereas more dominant girls were less popular.

Several studies indicate that there might be a connection between popularity and prosociality in children. When observing interactions between characters in video sequences, preschool children expect an individual with high regard from the other characters to help more in reaching a joint goal, but at the same time refrain from taking more than their relative proportion from a common resource (Stavans and Diesendruck, 2021). In line with these findings, preschoolers whose popularity was determined via high levels of visual regard from their peers had a higher tendency of choosing prosocial reward distributions in a resource sharing task with a familiar peer (Horn et al., 2024b). Moreover, 8–12-year-old children with high levels of visual regard were found to be particularly prosocial by donating resources and intervening in fights on behalf of the losing opponent (Ginsburg and Miller, 1981). Conversely, in a resource sharing experiment with 6–9-year-old children whose popularity was first measured by counting the number of interaction partners during a group observation period, children with low popularity preferred prosocial resource distributions, both when this choice benefitted another peer and when they were tested alone in a control situation (Horn et al., 2018). This highlights that the connection between popularity and children's prosocial behavior still needs to be investigated in more detail. Particularly the question whether a recipients' popularity influences the likelihood of becoming a target of prosocial actions remains under-researched.

### 1.3 Friendship and prosociality

Bronfenbrenner and Morris (2007) describe friendship as an emotional relationship between two individuals, that also shapes a developing individual's surroundings and is part of the way their surroundings are being perceived. Friendship formation occurs early in childhood and early friendships are characterized by spatial proximity and frequent social interactions (Bagwell and Bukowski, 2018). Friendships seem to mediate children's prosocial behavior. Children expect more prosocial acts such as sharing or helping between individuals who are friends than those who are not (Afshordi, 2019; Liberman and Shaw, 2017). Anonymized sharing studies, in which no peer is present but valuable items are said to be delivered to a specific other child, suggest that preschoolers are more likely to share with friends than peers that are not their friends. Paulus and Moore (2014) found that 3–5-year old preschoolers expected sharing to occur more often toward a friend than a disliked peer in an individual story-telling task and also shared more with a toy figure used as a stand-in for a friend than with a toy representing a disliked peer. In a setting, where an experimenter asked 3- and 5-year-olds to share stickers with a friend from their kindergarten group, a hypothetical child who would join the kindergarten group the next day or a hypothetical stranger, participants of both age groups gave more stickers to their friends than to any other category, regardless of whether they expected their prosocial act to be reciprocated or not (Lenz and Paulus, 2021). However, Berndt (1981) found that while children claimed they would share more with friends than acquaintances when being asked, actual sharing behavior did not differ between friends and non-friends when the children were paired with familiar peers in direct interactions. Several other studies in which preschoolers were tested in dyadic direct interactions with familiar peers similarly failed to find preferential sharing with friends (e.g., Horn et al., 2024b; Messer et al., 2017). Helping, on the other hand, seems to be influenced by friendship, even when looking at direct interactions between peers. In a forced-choice task conducted in triads of familiar peers, 3-year-old children preferentially provided help to the peer they had previously identified as their friend in a sociometric interview than toward the other, neutral peer from their kindergarten group (Engelmann et al., 2019). The same children also provided more help toward a friend in a second task, where they were paired either with a friend or a neutral peer from their group (i.e., children cleaned up a larger amount of paper shreds for their friends; Engelmann et al., 2019). These findings imply that some relational factors might affect preschoolers' sharing and helping behavior differently, indicating that it would be valuable to test and compare these two types of prosocial behavior and investigate how they are used among familiar preschool peers.

### 1.4 The present study

Existing research suggests that young children's dominance, popularity, and friendships influence their prosocial behavior. However, most of this evidence comes from laboratory studies testing children individually or in dyads. Only few studies have investigated how children use prosocial behavior in groups of familiar peers (e.g., Charlesworth and La Freniere, 1983), where interaction partners have pre-existing relationships that guide not only children's decisions about whether to help and share or not, but also which group members to direct these prosocial actions to. Moreover, while most studies and theoretical models placed a strong focus on actor characteristics predicting prosocial behavior, few studies have taken into account whether the recipient's characteristics might also modulate an acting child's prosocial behavior (e.g., Charafeddine et al., 2016; Paulus and Moore, 2014). Therefore, it remains unclear how prosocial decisions are made in preschoolers' natural environment—their peer group *microsystem* (Bronfenbrenner and Morris, 2007)—where dominance, popularity, and friendship are all important aspects of their everyday social life.

Guided by a developmental-ecological stance, the aim of the current study was to assess preschoolers' prosocial behavior, as well as their dominance, popularity, and friendships in groups of familiar peers and test how actor and recipient characteristics influence sharing and helping behavior. By testing participants in their respective preschools, in their socio-ecological niches with familiar peers, and in naturalistic settings, our research was conducted in an ecologically valid environment (cf. Bronfenbrenner, 1974; Read and Szokolszky, 2018). We first assessed peer friendship with age-appropriate sociometric interviews. In group game settings, we tested dominance in a contest and a scramble competition situation, as well as two types of prosocial behavior (i.e., sharing and helping). We chose games because games are a familiar part of preschoolers' everyday lives and we could at the same time carefully design these games to assess dominant and prosocial behavior. As external measures for dominance, popularity, and prosociality, we handed out questionnaires to preschool teachers. At each point during our age-appropriate experimental games, children could freely choose not only whether they wanted to engage in prosocial behavior or not, but also who would (or would not) become the target of their prosocial actions.

In the first step, we examined the predictors of children's dominance in peer groups. We hypothesized that dominance would increase with age (Hawley, 2002). We further hypothesized that there would be an interaction effect of popularity and gender, with more popular girls being less dominant and more popular boys being more dominant (Sebanc et al., 2003). For our main research question, we examined whether dominance and popularity of the actor and the recipient, as well as friendship between the children predicted sharing and helping. We further controlled for effects of actors' and recipients' gender and age. Considering actor characteristics, we expected higher dominance to decrease children's sharing and helping behavior (Guinote et al., 2015; Grueneisen and Tomasello, 2017). We further predicted that higher popularity would be associated with more sharing and helping (Ginsburg and Miller, 1981; Horn et al., 2024b) and that the children would display more prosocial actions toward friends than toward children who were not their friends in the group setting (Engelmann et al., 2019; Lenz and Paulus, 2021). We assumed that there would be an effect of gender, with girls being more prosocial, as well as an effect of age, with older children sharing and helping more often (for a meta-analysis, see Fabes and Eisenberg, 1998). Regarding recipient characteristics, there was very little literature from which we could derive our hypotheses. Due to popular children's central position in the social network, we expected higher recipient popularity to favor becoming the target of sharing and helping (McDonald and Asher, 2018). Similarly, we predicted higher dominance to favor becoming the target of prosocial actions among preschoolers (Charafeddine et al., 2016). We could not formulate clear hypotheses regarding the influence of recipient age or gender on becoming the target of prosocial actions, but chose to explore these factors in our analyses.

## 2 Materials and methods

### 2.1 Study design

The study consisted of three parts: (1) a sociometric assessment of popularity and friendship within the peer group, (2) three experimental sessions with age-appropriate games that were designed to elicit dominant and/or prosocial behavior in a group context, and (3) a teacher questionnaire, designed to provide external validity of children's popularity, friendships, dominance, and prosociality. The sociometric assessment was always conducted one day before the first experimental session. The experimental sessions were conducted 4–14 days apart (*Mdn* = 7). Each experimental session lasted ~30 min and consisted of three games. In the first game, designed as a contest competition, children could show dominance by gaining control over a limited resource (i.e., one novel toy that could only be used by one child at a given time). They could also show prosociality by passing the toy to another peer (i.e., “sharing”). In the second game, we measured dominance in a scramble competition. Children had to queue to obtain stickers of varying desirability and could show dominance by taking positions at the front of the queue. The third game was a freeze-unfreeze game, where children could show prosociality by “unfreezing” a peer, thereby allowing that peer to resume playing the game (i.e., “helping”). Teachers were asked to name a maximum of five best friends per child and rated each child's perceived prosociality and dominance.

### 2.2 Participants

In total, 108 3–6-year-old children from 14 preschool classrooms from five different childcare facilities in Vienna participated in the study (53 females, 55 males; age: *M* ± *SD* = 60.61 ± 11.47 months, *min* = 38, *max* = 80). There was no significant age difference between females and males [Welch's *t*-test: *t* (105.72) = 1.74, *p* = 0.084; females: *M* ± *SD* = 62.75 ± 10.68 months; males: *M* ± *SD* = 59 ± 11.68 months]. The children were tested in 11 groups of familiar peers, consisting of 6 to 13 children each (*Mdn* = 11). All children aged 3–6 years of the 14 preschool classrooms were invited to participate in the study. Final group size and composition of the tested groups depended on parental consent for participating in the study. The majority of groups was composed of children that attended the same preschool classroom with three exceptions: due to a low number of participants from six classrooms, we combined children from two classrooms that knew each other well and regularly interacted with each other into three suitable peer groups, respectively (i.e., groups C, I, and J). Due to individual absences, not all children took part in all sessions. One hundred children participated in the sociometric assessment and at least one of the game sessions. Two additional children did not participate in the sociometric assessment, but participated in at least one game session. Of these, 63 children participated in all three game sessions. Six additional children participated only in the sociometric assessment.

### 2.3 Sociometric assessment of popularity and friendship

To assess popularity and friendship, children participated in two rounds of sociometric assessment [adapted from Birch and Billman (1986)]. We chose a sociometric approach, because it provided an easy but reliable and age-appropriate measure for peer relationships in children. Each child was tested individually in a separate room at their childcare facility. To ensure peer recognition, we first showed the participant portrait photos of each other participating child from their group and asked them to name the peer depicted in the photo. In the first round, the participants were asked to assign each photo individually to one of three categories indicated by colored faces (green smiling face = “I like to play with this child,” red frowning face = “I do not like to play with this child,” yellow neutral face = “I do not know/care, whether I like to play with this child”). In the second round, the experimenter shuffled the peers' photos and handed them to the participant again. The participant was then asked to assign all photos corresponding to a specific category to one of three piles next to the three respective faces, until each photo was assigned to a category. During all instructions, the word “friend” was omitted to avoid socially desired answers (e.g., the participants stating that all peers are their friends). Overall, children showed substantial agreement in their ratings over the two rounds of the sociometric assessment (weighted κ = 0.69). In the various groups, agreement ranged from moderate to perfect (*M* ± *SD* = 0.67 ± 0.15, *min* = 0.41, *max* = 0.96).

To assess popularity, we used a standard procedure (e.g., Birch and Billman, 1986; Horn et al., 2024b; Ladd and Coleman, 1997) where we calculated each participant's acceptance and rejection scores by first counting the number of green and red nominations, respectively, that they had received from all other group members during the sociometric assessment. We then standardized the score by group size by dividing it by the number of all group members who rated the participant (i.e., group size −1). The minimum reachable score was 0 (i.e., no nominations by other children in the respective category) and the maximum was 1 (i.e., all nominations were green or red, respectively). Acceptance scores ranged from 0.05 to 1 (*M* ± *SD* = 0.47 ± 0.20) and rejection scores ranged from 0 to 0.85 (*M* ± *SD* = 0.24 ± 0.18). Both scales showed good to excellent internal consistency between first and second round (acceptance score: Cronbach's α = 0.90; rejection score: α = 0.88). Acceptance and rejection scores were moderately negatively correlated (*r* = –0.64, *p* < 0.001). Due to slightly higher internal consistency and broader range, we chose the acceptance score as our main variable for popularity. Females received significantly higher popularity scores than males (*t* (99.98) = 2.16, *p* = 0.041; females: *M* ± *SD* = 0.51 ± 0.19, males: *M* ± *SD* = 0.43 ± 0.20).

As friend nominations (*N* = 379) we counted those instances where a participant had allocated a peer to the green face in both rounds. All other nominations (*N* = 623) were summarized in a “non-friend” category. Children reported having between 0 and 12 friends (*Mdn* = 3). There was no difference in the number of friends reported by females and males (*t*(103.78) = 0.23, *p* = 0.819).

### 2.4 Game 1: contest competition and sharing game

The first experimental game was designed as a contest competition over a single valuable resource (cf. Pellegrini, 2008), which is a widely used method to assess dominance in social dominance research (Charlesworth and La Freniere, 1983; Grueneisen and Tomasello, 2017). In our game, children got a limited amount of time to play with a novel toy that could only be used by one participant at a time (3 min for groups with <10 participants, 5 min for groups with ≥10 participants). Children sat down in a circle and the experimenter explained that they would get to play with a toy for a certain amount of time. The experimenter explicitly stated that she or he would not interfere during that time, meaning that children had to settle toy possession themselves. When all children agreed to having understood the instructions, the experimenter placed the toy in the middle of the circle and turned around an hourglass indicating the duration of the game. The experimenter informed the children when half the time had passed. At the end of the game, the experimenter collected the toy and put it out of sight. A different toy was used in each of the three experimental sessions. All instructions were given without using the word “share,” to avoid socially desired sharing behavior.

We recorded the total duration of resource possession of each child (i.e., the total duration that the toy was in a specific child's hands during the game). For those children that participated in all three game sessions, resource possession was correlated across all sessions (Spearman's rank correlation: *n* = 63; sessions 1 and 2: ρ = 0.45, *p* < 0.001; sessions 1 and 3: ρ = 0.28, ρ = 0.029; sessions 2 and 3: *r* = 0.54, *p* < 0.001), indicating good internal consistency of this variable. We also recorded the sequence in which the children obtained possession of the toy as the contest rank (i.e., rank 1 = had the toy first, rank 2 = had the toy second, etc.). If a child took possession of the toy more than once, this respective rank was skipped and the next-higher rank was assigned to the child that got possession afterwards. We then standardized the contest rank by group size by dividing it by the number of participants in the respective session. Children who never took possession of the toy did not receive a contest rank score in this session and were omitted from analyses including this variable (session 1: *n* = 5; session 2: *n* = 19; session 3: *n* = 14). Contest rank was not significantly correlated across sessions (*n* = 40; sessions 1 and 2: ρ = 0.25, *p* = 0.124; sessions 1 and 3: ρ = 0.08, *p* = 0.623; sessions 2 and 3: ρ = 0.08, *p* = 0.614) and was therefore dropped from further analyses due to insufficient internal consistency. As a variable for prosociality, we counted all instances of sharing (i.e., passing the toy to another child). Sharing was correlated across all sessions (*n* = 63; sessions 1 and 2: ρ = 0.48, *p* < 0.001; sessions 1 and 3: ρ = 0.47, *p* < 0.001; sessions 2 and 3: ρ = 0.51, *p* < 0.001), indicating good internal consistency of this variable.

### 2.5 Game 2: scramble competition game

The second experimental game was designed as a scramble competition (cf. Pellegrini, 2008), where all children received stickers of varying desirability, but by having to queue, some children got access to the stickers earlier than others (adapted from the ticket paradigm; Pellegrini et al., 2007). While seated in the circle, the experimenter read a picture book in which animal characters had to queue to receive stickers. The first animal in the queue could choose the “coolest” sticker, while the last in the queue had to take a “boring” sticker. Each child was asked two comprehension questions (i.e., “Who can choose a sticker first?”, “Who is the last one to choose a sticker?”). Thereafter, the experimenter told the children that they would have 1 min to form a queue to receive stickers themselves and showed them a box with stickers of varying desirability. The experimenter explicitly stated that she or he would not interfere during that time, meaning that children had to settle their positions in the queue themselves. Upon the experimenter's signal, the children got up and had 1 min to form a queue. When the queue was final, children could approach the experimenter one-by-one and each choose one sticker from the box.

We used the position in the queue to determine each child's scramble rank as a variable for dominance, with lower numbers indicating a better position in the cue and therefore higher dominance. We then standardized the scramble rank by group size by dividing it by the number of participants in the respective session. If a child failed to answer one or both of the comprehension questions correctly, their position in the queue was considered when assigning other children's scramble rank, but they did not receive a scramble rank score in this session and were omitted from analyses including this variable (session 1: *n* = 15; session 2: *n* = 6; session 3: *n* = 3). For those children that received a scramble rank in all three game sessions, scramble rank was correlated between sessions 2 and 3 (Spearman's rank correlation: *n* = 48; ρ = 0.51, *p* < 0.001), but not between sessions 1 and 2 (ρ = 0.22, *p* = 0.141) or sessions 1 and 3 (ρ = 0.04, *p* = 0.796), indicating limited internal consistency of this variable.

### 2.6 Game 3: helping game

The third experimental game was a “freeze-unfreeze” game that the children played for 5 min. The aim of the game was to balance a plush toy on the head while moving to music. If the toy dropped from a child's head, the child had to “freeze” and stop moving. Another peer could pick up the toy and place it back on the child's head to “unfreeze” them, thereby allowing the child to resume playing the game. The game was designed to not produce any winners, so that children would not have to compete with one another. Two experimenters demonstrated the rules of the game to the children. All instructions were given without using the word “help,” to avoid socially desired helping behavior. When all children agreed to having understood the instructions, they were allowed to choose a plush toy. The experimenter then started the music and told the children that they could play the game until the music ended. If children were so good at balancing the toys that none dropped from their heads, the experimenter suggested “challenges,” such as walking backwards. After the music ended, the experimenter collected the plush toys and children were thanked for participating in this session and sent back to their classrooms.

We recorded all instances of a child picking up another child's plush toy. Instances of helping were correlated across all sessions (Spearman's rank correlation: *n* = 63; sessions 1 and 2: ρ = 0.58, *p* < 0.001; sessions 1 and 3: ρ = 0.64, *p* < 0.001; sessions 2 and 3: ρ = 0.60, *p* < 0.001), indicating good internal consistency of this variable.

### 2.7 Teacher questionnaire assessing popularity, friendship, dominance, and prosociality

As external measures for children's popularity and friendship, we asked teachers to name a maximum of five best friends from all participating peers for each participant. This method was chosen to ensure comparability to children's ratings while reducing time and effort for the teachers. The teachers also rated each child's perceived dominance and prosociality on a continuous, graphic rating scale ranging from “strongly disagree” to “strongly agree.” There were four items for dominance (i.e., “assertive,” “dominates classmates,” “tells others what to do,” “stands up for self”; Pellegrini et al., 2011) and four items for prosociality (i.e., “helps other children,” “willingly shares with other children,” “offers assistance and comfort to other children,” “seems to be moved by other children's distress”; Ladd and Profilet, 1996). Per preschool classroom, the questionnaire was filled out by two teachers that knew all participants well, with two exceptions: for groups C and E, we could only acquire a single teacher questionnaire.

Popularity was measured by counting how often a child had been nominated as a friend of another child. As with children's nominations, we standardized the popularity score by group size by dividing the score by the number of other group members. Teacher-rated popularity scores ranged from 0 to 0.83 (*M* ± *SD* = 0.32 ± 0.20). Overall, teachers' agreement on the popularity scores was high (Cronbach's α = 0.82). For further analyses, a mean popularity score over all raters was calculated for each child. For friend nominations, agreement between teachers was overall moderate (Cohen's κ = 0.58) and ranged from slight to perfect in the various groups (*M* ± *SD* = 0.50 ± 0.26, *min* = 0.12, *max* = 1). Due to the very low consistency of teacher's friendship nominations for some of the groups, we did not use the friendship nominations for external validity.

Ratings on the continuous graphic rating scale were measured with a ruler and standardized, so that the range of possible values was between 0 and 10. Dominance and prosociality ratings showed good to excellent internal consistency (dominance: α = 0.91; prosociality: α = 0.88). Therefore, for each teacher, a mean rating per child was calculated over the four items of dominance and prosociality, respectively. Agreement between raters was high for dominance (α = 0.84) and acceptable for prosociality (α = 0.73). Dominance ratings ranged between 0.10 and 9.89 (*M* ± *SD* = 5.14 ± 2.56) and prosociality ratings ranged between 0.10 and 9.80 (*M* ± *SD* = 5.79 ± 2.36). For further analyses, a mean dominance and prosociality score over all raters was calculated for each child.

### 2.8 Data analyses

All hypotheses and predictions were formulated before data collection started, but they were not pre-registered online. No additional data were collected after the analyses. Sample size estimation was conducted with G*power (Faul et al., 2007) and based on the linear models in the first part of our analysis (see details below). Sample size estimation for negative binomial mixed models was not available to the authors at the time of conceiving this study. Sample size estimation for linear models with five predictors yielded a sample size of 92 in order to detect medium effect sizes (*f*^2^ = 0.15; α err prob = 0.05; 1–β err prob = 0.80). Since based on our previous work in childcare facilities we expected an attrition rate of about 15% between children that received parental consent for participation and those that were actually able to participate in the experimental sessions, we decided to recruit 108 participants. Actual attrition was lower than expected at 5.6% for the main analysis. Therefore, the two linear models were slightly over-powered compared to the sample size estimation. Sample size estimation for bivariate correlations yielded a minimum sample size of 67 in order to detect correlations of medium strength (ρ = 0.3; α err prob = 0.05; 1–β err prob = 0.80). Sample sizes were large enough to meet these criteria in all main analyses. Sample sizes were not large enough when conducting the internal consistency checks of the behavioral variables, since they were conducted with data obtained only from children that were present in all three game sessions (range of *n* = 40–63). *Post hoc* analyses revealed that power to detect significant correlations of medium strength was only 1–β err prob = 0.60–0.78 for these internal consistency checks. Statistical analyses were carried out in R version 4.2.1 (R Core Team, 2022). *P*-values < 0.05 were considered statistically significant and *p*-values < 0.1 are reported as non-significant trends. Due to the tested groups' similarity in gender distribution (χ^2^ = 16.52, *df* = 10, *n* = 108, *p* = 0.086), age (Kruskal–Wallis: χ^2^ = 12.593, *df* = 10, *n* = 108, *p* = 0.247), and number of friends (Kruskal–Wallis: χ^2^ = 10, *df* = 10, *n* = 108, *p* = 0.441), we used the full sample for statistical analyses and did not include group as a predictor.

To assess construct and external validity, we calculated mean values per child for each variable and compared them to each other and to teachers' ratings, respectively. To assess construct validity for dominance and prosociality, respectively, we calculated Spearman's rank order correlations with duration of resource possession in game 1 and position in the queue in game 2 as well as sharing in game 1 and helping in game 3. To test external validity for dominance, we correlated teacher rated dominance with children's duration of resource possession with a Pearson correlation and children's scramble rank with a Spearman's rank order correlation, respectively. Similarly, we assessed external validity for prosociality by performing a Spearman's rank order correlation for teacher rated prosociality and children's sharing in game 1 and helping in game 3, respectively. External validity for popularity was examined by calculating a Pearson correlation for popularity determined via friendship nominations made by teachers and children themselves. Not all children participated in all parts of this study and/or obtained a score in each of the experimental games (see previous sections). We conducted each correlation with the maximum sample size possible for the respective pair of variables.

To test which factors influence children's overall dominance, we first calculated a mean of the variables duration of resource possession in game 1 and scramble rank in game 2 across all three experimental sessions, respectively. Further, we did a logarithmic transformation of the two variables for dominance, as the data did not follow a normal distribution. We then fitted two linear models with outcome variables duration of resource possession and scramble rank, respectively. Predictor variables were popularity, gender, their interaction, age, and group size.

To test how individual characteristics of the actor and recipient influenced each occurrence of prosocial behavior, we fitted two negative binomial mixed models. Outcome variables were the occurrences of sharing in game 1 and helping in game 3, respectively. Predictors were friendship between interaction partners, as well as dominance, popularity, gender, age of the actor and the recipient of the prosocial action, respectively. As predictor variable for dominance, we chose children's duration of resource possession in game 1 of the respective session, because this variable had greater internal consistency and fewer missing values than scramble rank. We controlled for group size and session by adding them as predictors. The individual was added as fixed effect.

All models were examined for goodness of fit by fitting simulated residuals against those predicted with the DHARMa package for R (Hartig, 2022). With the same package, we did a Kolmogorov–Smirnov–Test, an outlier test and a dispersion test for each model. Models were considered to have a good fit, if no significant deviations were detected in the residuals plot or any of the tests.

## 3 Results

When testing the construct validity of the different measures of dominance and prosociality, respectively, we found that children's duration of resource possession and scramble rank were weakly negatively correlated (Spearman's rank correlation: *n* = 99; ρ = −0.31, *p* = 0.002), meaning that children who were in possession of the toy longer in game 1 also took positions in the front of the queue in game 2. Similarly, children who shared more in game 1 also helped more in game 3 (*n* = 102; ρ = 0.28, *p* = 0.006). Compared to the teacher questionnaires, we found that teachers' dominance ratings were weakly correlated with both, duration of resource possession (Pearson correlation: *n* = 102; *r* = 0.22, *p* = 0.027) and scramble rank (*n* = 99; ρ = –0.27, *p* = 0.008). However, there was no correlation between teachers' prosociality rating and either children's sharing in game 1 (*n* = 102, ρ = –0.12, *p* = 0.248) or their helping in game 3 (*n* = 102; ρ = 0.05, *p* = 0.598). Teachers' popularity ratings were moderately positively correlated with popularity scores obtained from children's sociometric assessments (*n* = 106; *r* = .48; *p* < 0.001).

**Figure 1:**
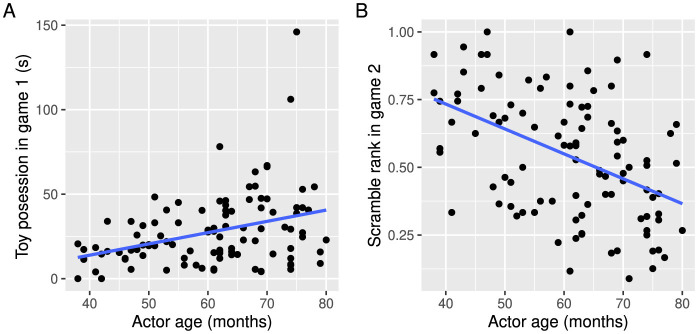
The effect of children's age on their dominance estimated via **(A)** duration of toy possession in game 1 and **(B)** scramble rank in game 2. The blue line shows the linear regression.

In our investigation of the factors affecting children's dominance, the first linear model revealed a significant regression for dominance estimated via duration of resource possession (*n* = 102; *R*^2^ = 0.09, *F*_(5, 96)_ = 2.97, *p* = 0.015; see Table 1 for detailed results). Specifically, duration of resource possession was positively predicted by age (Figure 1A), meaning that older children were in possession of the toy longer in game 1. The linear model for dominance estimated via scramble rank also revealed a significant regression (*n* = 102; *R*^2^ = 0.25, *F*_(5, 93)_ = 6.06, *p* < 0.001; see Table 2). Scramble rank was negatively predicted by age, meaning that older children had access to the stickers sooner in game 2 (Figure 1B). None of the other predictors were significant in the two models (Tables 1, 2).

**Table 1:** Results of the linear model for dominance estimated via duration of resource possession in game 1.

**Parameter**	**Estimate**	**SE**	**CI lower**	**CI higher**	***t* (96)**	***p***	**ω^2^*^a^***
(Intercept)	0.91	0.67	–0.42	2.23	1.36	0.177	
Popularity	0.31	0.63	–0.94	1.57	0.50	0.622	0.00
Gender (M)	–0.00	0.45	–0.88	0.88	–0.00	0.998	0.00
Popularity*gender (M)	0.29	0.88	–1.45	2.03	0.33	0.742	0.00
Age (months)	**0.03**	**0.01**	**0.012**	**0.041**	**3.577**	**<0.001**	**0.11**
Group size	0.03	0.04	–0.047	0.107	0.782	0.436	0.00

Given are estimates, standard errors (SE), confidence intervals (CI lower, CI higher), *t*-values, *p*-values, and partial omega squared (ω^2^) for the intercept and each predictor of the model.

Predictors with *p*-values ≤ 0.05 are highlighted in bold.

a Effect size is partial omega squared (ω^2^; Keppel, 1991; large: ω^2^ > 0.14, medium: ω^2^ = 0.06 − 0.14, small: ω^2^ = 0.01 − 0.06).

**Table 2:** Results of the linear model for dominance estimated via scramble rank in game 2.

**Parameter**	**Estimate**	**SE**	**CI lower**	**CI higher**	***t* (96)**	***p***	**ω^2^ *^a^***
(Intercept)	**0.97**	**0.38**	**0.23**	**1.72**	**2.59**	**0.011**	
Popularity	–0.37	0.35	–1.07	0.33	–1.05	0.298	0.00
Gender (M)	–0.06	0.25	–0.57	0.44	-0.24	0.811	0.00
Popularity*gender (M)	0.00	0.50	–0.99	0.99	0.01	0.995	0.00
Age (months)	**–0.02**	**0.00**	**–0.03**	**-0.01**	**–5.15**	**<0.001**	**0.21**
Group size	–0.02	0.02	–0.06	0.03	–0.72	0.473	0.00

Given are estimates, standard errors (SE), confidence intervals (CI lower, CI higher), *t*-values, *p*-values, and partial omega squared (ω^2^) for the intercept and each predictor of the model.

Predictors with *p*-values ≤ 0.05 are highlighted in bold.

a Effect size is partial omega squared (ω^2^; Keppel, 1991; large: ω^2^ > 0.14, medium: ω^2^ = 0.06 − 0.14, small: ω^2^ = 0.01 − 0.06).

**Figure 2:**
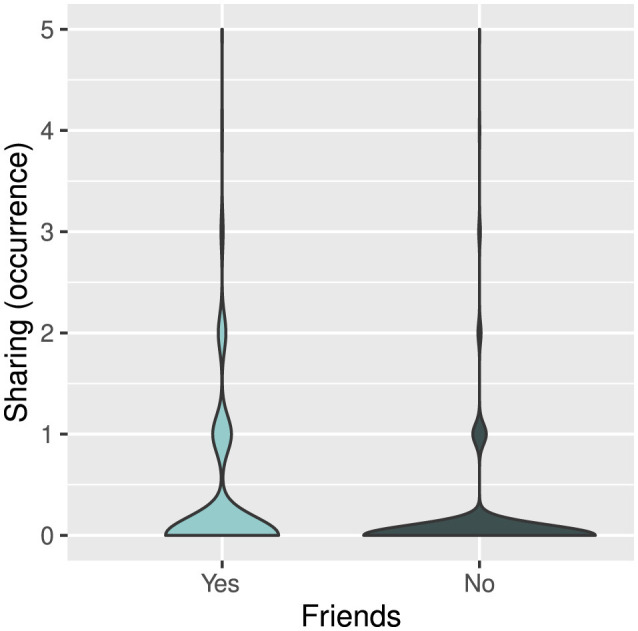
Occurrences of sharing with friends (lightblue) and other children (darkblue). The width of each density curve corresponds with the approximate frequency of data points in each region.

**Figure 3:**
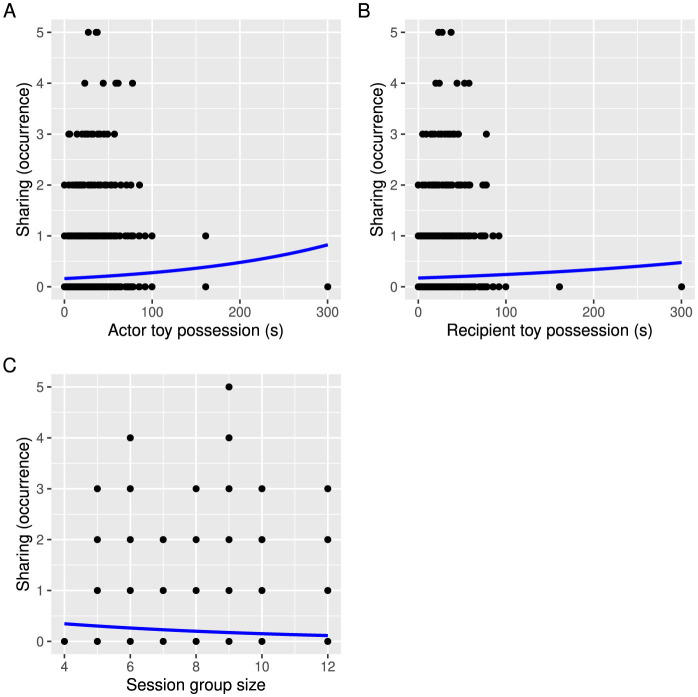
The effects of **(A)** actor children's dominance and **(B)** recipient children's dominance estimated via duration of toy possession in game 1, as well as **(C)** session group size on the occurrences of sharing in game 1. The blue line shows the poisson regression.

When investigating which actor and recipient characteristics predicted children's prosociality, we found that sharing in game 1 was significantly positively predicted by friendship as well as actors' and recipients' dominance as measured by their toy possession, and negatively by group size (Table 3). Children were more likely to share the toy with friends than with children who were not their friends (Figure 2). Children that were able to hold on to the toy longer in game 1 were more likely both to share the toy (Figure 3A) and to receive the toy from others (Figure 3B). Group size negatively affected sharing, meaning that there was less sharing in larger groups (Figure 3C). None of the other predictors were significant (Table 3).

**Table 3:** Results of the mixed model for sharing in game 1 (*n* = 100; number of observations = 1,697).

**Parameter**	**Estimate**	**SE**	**CI lower**	**CI higher**	***z***	***p***	**OR*^b^***
(Intercept)	–0.80	0.84	–2.45	0.86	–0.94	0.345	0.45
Friends	**1.11**	**0.17**	**0.77**	**1.45**	**6.46**	**<0.001**	**3.03**
Actor toy possession	**0.01**	**0.00**	**0.00**	**0.01**	**2.78**	**0.005**	**1.01**
Recipient toy possession	**0.01**	**0.00**	**0.00**	**0.01**	**2.23**	**0.025**	**1.01**
Actor popularity	0.03	0.45	–0.85	0.90	0.06	0.953	1.03
Recipient popularity	–0.57	0.45	-1.45	0.31	–1.27	0.204	0.57
Actor gender (M)	–0.07	0.17	–0.40	0.26	–0.40	0.691	0.93
Recipient gender (M)	0.03	0.16	-0.28	0.35	0.20	0.838	1.03
Actor age (months)	–0.00	0.01	–0.02	0.01	–0.15	0.882	1.00
Recipient age (months)	–0.01	0.01	–0.02	0.01	–1.19	0.233	0.99
Session [2]	–0.02	0.19	–0.39	0.34	–0.11	0.911	0.98
Session [3]	–0.09	0.19	–0.46	0.28	–0.48	0.628	0.91
Session group size	**–0.13**	**0.05**	**–0.22**	**–0.04**	**–2.73**	**0.006**	**0.88**

Given are estimates, standard errors (SE), confidence intervals (CI lower, CI higher), *z*-values, *p*-values, and odds ratios (OR) for the intercept and each predictor of the model. Predictors with *p*-values ≤ 0.05 are highlighted in bold.

b Effect size is the odds ratio; the larger the odds ratio, the greater the effect (Rosnow and Rosenthal, 2003).

**Figure 4:**
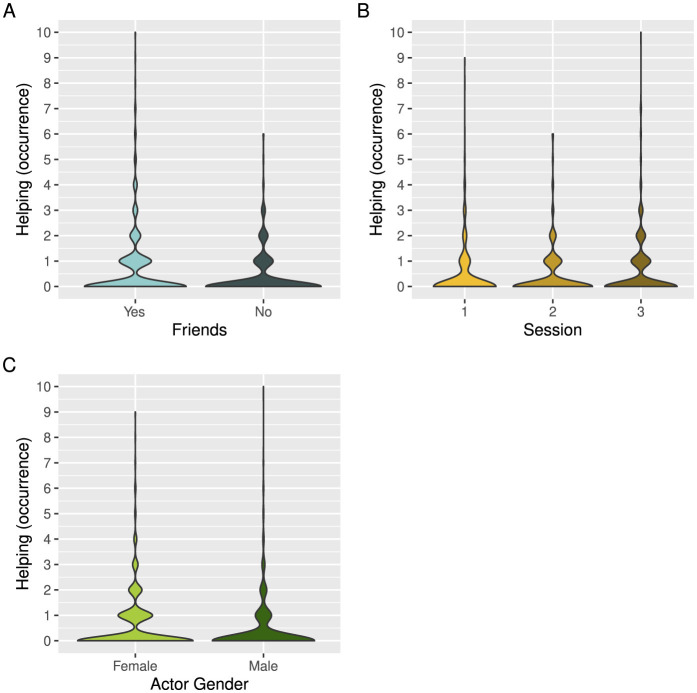
Occurrences of helping when comparing **(A)** friends (lightblue) and other children (darkblue), **(B)** across sessions 1–3 (orange, lightbrown, darkbrown), and **(C)** female participants (lightgreen) and male participants (darkgreen). The width of each density curve corresponds with the approximate frequency of data points in each region.

**Figure 5:**
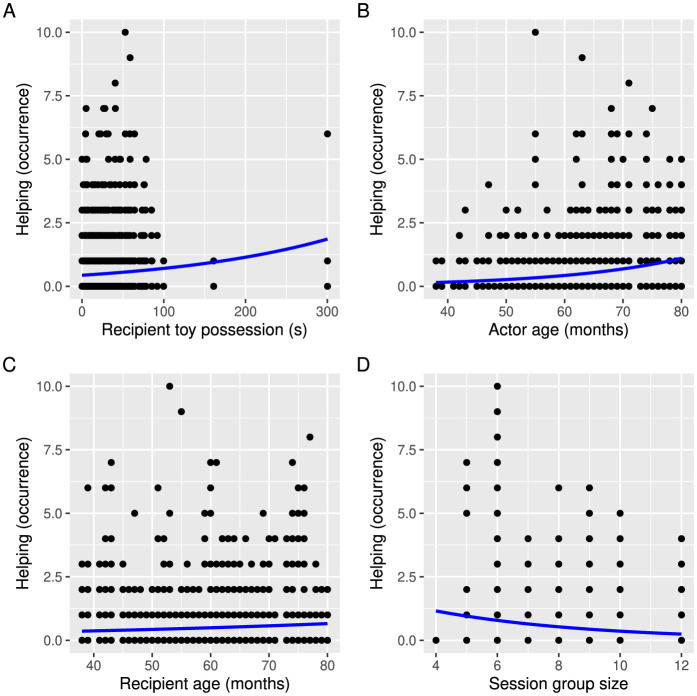
The effects of **(A)** recipient children's dominance estimated via duration of toy possession in game 1, **(B)** actor children's age, **(C)** recipient children's age, and **(D)** session group size on the occurrences of helping in game 3. The blue line shows the poisson regression.

Helping in game 3 was significantly predicted by friendship, recipient dominance as measured by toy possession in game 1, actor and recipient age, as well as session and group size (Table 4). Children were again more likely to help friends than children who were not their friends (Figure 4A). The longer the children were able to hold on to the toy in game 1, the more likely they were to receive help in game 3 (Figure 5A). Further, the older the acting children and the recipient children were, the more likely they were to help and receive help, respectively (Figures 5B, C). Children were more likely to help in the third session compared to the first session (Figure 4B). Group size negatively affected helping, meaning that there was less helping in larger groups (Figure 5D). Additionally, there was a non-significant trend that female participants were more likely to help than male participants (Figure 4C). None of the other predictors were significant (Table 4).

**Table 4:** Results of the mixed model for helping in game 3 (*n* = 100; number of observations = 1,697).

**Parameter**	**Estimate**	**SE**	**CI lower**	**CI higher**	***z***	***p***	**OR*^b^***
(Intercept)	**–4.97**	**0.85**	**–6.60**	**–3.30**	**–5.86**	**<0.001**	**0.01**
Friends	**0.46**	**0.11**	**0.24**	**0.67**	**4.16**	**<0.001**	**1.58**
Actor toy possession	–0.00	0.00	0.00	0.00	–0.05	0.957	1.00
Recipient toy possession	**0.00**	**0.00**	**0.00**	**0.01**	**3.30**	**0.001**	**1.00**
Actor popularity	0.32	0.53	–0.70	1.36	0.59	0.552	1.37
Recipient popularity	–0.28	0.30	–0.90	0.32	-0.92	0.357	0.76
*Actor gender (M)*	–*0.37*	*0.21*	–*0.80*	*0.04*	–*1.79*	*0.073*	*0.69*
Recipient gender (M)	0.17	0.10	0.00	0.36	1.64	0.102	1.18
Actor age (months)	**0.06**	**0.01**	**0.04**	**0.08**	**6.14**	**<0.001**	**1.06**
Recipient age (months)	**0.01**	**0.00**	**0.01**	**0.02**	**3.22**	**0.001**	**1.01**
Session [2]	-0.10	0.12	–0.30	0.14	–0.80	0.423	0.91
Session [3]	**0.30**	**0.12**	**0.07**	**0.54**	**2.50**	**0.013**	**1.35**
Session group size	**–0.13**	**0.04**	**–0.20**	**–0.05**	**–3.08**	**0.002**	**0.88**

Given are estimates, standard errors (SE), confidence intervals (CI lower, CI higher), *z*-values, *p*-values, and odds ratios (OR) for the intercept and each predictor of the model. Predictors with *p*-values ≤ 0.05 are highlighted in bold. Predictors with *p*-values < 0.1 are highlighted in italics.

b Effect size is the odds ratio; the larger the odds ratio, the greater the effect (Rosnow and Rosenthal, 2003).

## 4 Discussion

The aim of this study was to examine children's prosocial behavior within a developmental-ecological framework. We assessed preschoolers' prosocial behavior, as well as their dominance, popularity, and friendships in ecologically valid, naturalistic group settings with familiar peers and tested how actor and recipient characteristics influenced children's sharing and helping. Children's dominance in games 1 and 2 was influenced by age, with older children being more dominant in both the contest and the scramble competition setting. Moreover, children who were in possession of the toy longer in game 1 also gained access to the stickers earlier in game 2. Teachers' dominance ratings were weakly correlated with both dominance variables but not with children's prosocial behaviors. Teachers' popularity ratings were moderately correlated with popularity scores obtained from children's sociometric assessments. When investigating children's prosocial choices, we found that children who shared more in game 1 also helped more in game 3. Both types of prosocial behavior were directed more often toward friends than toward children that were not considered as friends. The likelihood of sharing in game 1 was higher both for actors and recipients that were in possession of the toy for longer durations. In game 3, recipients that had possessed the toy longer in game 1 were also more likely to receive help. Further, both actor and recipient age increased the likelihood of helping in game 3. Additionally, we found that children helped more often in the final than in the first experimental session and there was a non-significant trend that females helped more often than males in game 3. In both games there was a negative effect of group size: the larger the groups were, the less likely individual children were to share and help. Actors' or recipients' popularity had no effect on both types of prosocial behavior.

The strongest predictor for prosocial behavior in our study was friendship. Friendship increased the likelihood of helping and sharing, which is in line with existing results from a helping study where familiar peers interacted directly (Engelmann et al., 2019) and from anonymized sharing studies where the recipients were represented by photos or puppets (Lenz and Paulus, 2021; Paulus and Moore, 2014). Direct-interaction sharing experiments where familiar peers were tested in dyads, on the other hand, had previously often failed to find more sharing with friends than non-friends in preschoolers (e.g., Horn et al., 2024b; Messer et al., 2017). It is possible that, in settings where children are not required to choose between different partners (cf. Engelmann et al., 2019) and are simply assigned to a dyad with a familiar peer, children share equally irrespective of their friendship status due to general fairness principles (Dunfield, 2014). Such fairness principles have been argued to emerge early in childhood, even if they are later shaped by children's experiences in their social environment (Geraci, 2022). In preschoolers' everyday interactions in their natural environment, children regularly have to make decisions not only whether to share or not, but also whom to share with. Therefore, the findings of the current study are likely more ecologically valid than dyadic experiments and add important new insights to the existing literature on how friendship shapes young children's prosocial behavior.

Other than by friendship, sharing in game 1 was mainly predicted by actors' and recipients' dominance, which we estimated via the duration that children were able to hold possession of the toy. Specifically, children that were in possession of the toy longer were more likely to share the toy and also to receive the toy from others. Although the former result seems to contradict our hypothesis that higher dominance would decrease children's sharing behavior (cf. Guinote et al., 2015), one must consider that our measures of dominance and prosocial behavior were likely codependent in game 1. Only children that were able to take possession of the toy for some of the time during game 1 were also able to share the toy with others. Similarly, children that held possession of the toy longer were more likely to have received it previously from one of their peers. Therefore, it is difficult to disentangle whether the observed effect in this analysis was due to children's dominance or due to the game affordances. Nevertheless, recipient toy possession in game 1 was also a positive predictor of helping in game 3, where no such codependency existed. Our result that higher dominance favors becoming the target of prosocial actions among preschoolers is in line with experimental findings showing that young children preferentially donate resources to a dominant puppet (Charafeddine et al., 2016). Overall, our results suggest that the association between dominance and prosociality in children's natural social environment is likely complex. While experimental studies in which children are tested individually or in dyads point to a negative association between children's dominance and their prosocial behavior when sharing resources (Guinote et al., 2015; Horn et al., 2024b), observations of preschoolers interactions in natural social groups indicate that the use of dominant and prosocial behavior can sometimes go hand in hand (Hawley, 1999). In her strategy-based evolutionary perspective on children's dominance, Hawley (1999) postulates that dominance relates to the ability to control resources—irrespective of how this is done—and that children employ different strategies to compete with peers (i.e., coercive and prosocial). In preschool age, some children have been found to use predominantly coercive or predominantly prosocial strategies to acquire resources, whereas so-called bistrategic controllers make use of both strategies, depending on the context (Hawley, 2003). Natural peer groups are typically composed of a mixture of coercive, prosocial, and bistrategic controllers (as well as non-controllers; Hawley, 2003). The association between dominant and prosocial behavior might be different, depending on which type of resource control strategy each child prefers. Coercive controllers will likely show the negative association with prosocial behavior by always withholding resources. In prosocial controllers, however, there might even be a positive association with prosocial behavior in natural interactions, as they typically acquire resources by using some form of prosocial strategy such as sharing a non-preferred toy in order to gain access to a preferred toy (Hawley, 1999). Therefore, in our natural group setting where multiple peers interacted with each other, it is possible that their different behavioral strategies obscured any clear association between dominance and prosociality. Future studies investigating the connection between prosociality and dominance should therefore not only assess dominance via children's ability to control resources, but also focus on the individual strategies used by children when competing with peers. These detailed observations of children's behavioral strategies are only possible in their natural social environment.

Apart from friendship and recipient dominance, which affected both sharing and helping, other factors only influenced helping behavior in our study. We found that older children not only helped more often, but also received help more often. A greater helping propensity in older children replicates well-established findings that prosociality generally increases as children get older and develop more sophisticated socioemotional skills (Fabes and Eisenberg, 1998; Flook et al., 2019). One reason for the effect that older children also received more help could be that preschool children in natural groups preferentially socialize with children that are similar to them (e.g., peers of the same gender or of similar age; Howes, 1983). In the helping game, the children were moving more freely than in the sharing game, where they were required to sit in a circle. Therefore, spatial proximity due to similarity might have led to older children spending more time close to each other in this game, which would have facilitated helping interactions between these older children. Alternatively, there might have been more reciprocation of prosocial actions in the helping game than in the sharing game in our study, although existing studies typically show more reciprocity of sharing than of helping behavior (Messer et al., 2017; Warneken and Tomasello, 2013). Moreover, we found that children helped more in the last session than in the first one, which could simply be attributed to the fact that they understood the game better as well as were more practiced in the task, allowing them to recognize others' need for help and to perform the helping action better. There was a non-significant trend that girls helped more than boys. Helping has been more strongly linked to empathy and social understanding (Hay, 2023), while sharing is thought to be more dependent on children's moral norms and fairness principles (Dunfield, 2014). Females, even at a young age, have often been found to be more empathic than males due to socialization (Fabes and Eisenberg, 1998) as well as biological determinants (Christov-Moore et al., 2014). These differences in empathic concern might explain why gender had a tendency to influence the likelihood of helping and why this tendency was present in the helping game, but not in the sharing game.

Our findings suggest that there are some similarities and some differences between sharing and helping at preschool age. Both prosocial behaviors were correlated, though only weakly, suggesting that preschool children already have some general prosocial tendencies (Knafo-Noam et al., 2015) and that both behaviors may have some basic underlying motivations, such as to alleviate somebody else's negative emotional state (Paulus, 2014, 2018). Nevertheless, the fact that we also found a number of factors that differently influenced sharing and helping, adds to the body of evidence suggesting that early prosociality is of a multidimensional nature and that sharing and helping are heterogeneous behaviors with different antecedents (Hay, 2023; Spinrad and Eisenberg, 2023). Until toddlerhood, sharing and helping have been argued to be related to different social-cognitive mechanisms and underlying motives (Paulus, 2014). While early sharing in young children seems to occur predominantly in response to other persons' explicit requests (Schmidt and Sommerville, 2011), studies on early helping behavior suggest that processes of goal contagion (Michael and Székely, 2019) or a motivation to engage with the other (Paulus and Moore, 2014) seem to underlie helping. Paulus (2018) postulates that, as children get older, general prosociality might get integrated into their personality as a coherent character trait (see also Knafo-Noam et al., 2015). The 3–6-year-old preschoolers tested in the current study might be in a transition phase, where distinct prosocial behaviors are only weakly correlated, but get more and more integrated as the children mature.

Contrary to our expectations, neither the actors' nor the recipients' popularity predicted sharing or helping in our study. Popularity measured via visual regard was positively associated with sharing in a dyadic experiment with a familiar peer (Horn et al., 2024b) and with intervening in fights during natural observations (Ginsburg and Miller, 1981). Conversely, 6–9-year-old children that were ascribed low popularity due to having few interaction partners have been found to prefer prosocial resource distributions in a dyadic experiment (Horn et al., 2018). Testing children in a group setting with familiar peers did not reproduce either effect, signaling that the connection between popularity and prosocial behavior is indeed more complex. Interestingly, we found that girls, who overall had a tendency for showing more helping behavior than boys, were also slightly more popular than boys. This might point to an indirect positive association between popularity and prosociality, mediated by gender. In fact, a desire to increase their reputation might have encouraged less popular individuals to behave prosocially more strongly in the group setting than when they are tested individually or in a dyad (cf. Engelmann et al., 2018). This might have masked a clear association between actors' popularity and sharing or helping. We did not confirm the predicted positive effect of recipients' popularity either. Due to popular children's central position in the social network, we would have expected higher recipient popularity to favor becoming the target of sharing and helping (Newcomb and Bukowski, 1983). It is possible, however, that other factors that were not investigated in this study are more relevant triggers for prosocial behavior in preschool children. For example, Geraci et al. (2021) found that preschoolers were particularly prosocial to an agent that showed comforting behavior to a victim in an experimental context, while the relationships between the observed agents seemed to be less relevant for their prosocial choices (for similar results in younger children, see also Geraci, 2020). Future studies should take into account additional person characteristics that might influence children's prosocial behavior beyond popularity, friendship, and dominance.

When examining our behavioral measures of dominance, we found that duration of resource possession and the ability to acquire a front position when queuing for stickers were weakly correlated. Moreover, both dominance variables were predicted by age in the same way. Building on behavioral ecological theory, Pellegrini (2008) argued that contest competition and scramble competition produced distinct patterns of social behavior in preschool children. However, our results suggest that the children either had a relatively stable position in the dominance hierarchy of their peer group or a general inclination to compete for resources and that they behaved accordingly even when tested in different competition contexts (Pellegrini et al., 2007, 2011). While we were able to replicate the well-established increase of dominance in a peer group with age (Hawley, 2002), we could not find the predicted interaction effect of gender and popularity (Sebanc et al., 2003). In Sebanc et al.'s (2003) study, more dominant boys were more popular among peers than less dominant boys, while more dominant girls were less popular. It has to be noted, though, that in this case popularity was assessed after children had finished all dominance tasks, so that popularity ratings might have been affected by children's dominant actions during the preceding experiments. In the current study, popularity ratings were collected prior to any experimental interactions between the children and therefore represented a more general measure for popularity. In future studies, it would be interesting to assess popularity both before and after dominance experiments to get a full picture of the connection between the expression of dominant behavior and popularity among peers. Another reason for not finding that dominant behavior was differently associated with popularity in the two genders might be that nowadays female dominance becomes more accepted in society and children might shape their opinions based on mothers and other female role models that openly express dominant behavior (Greene et al., 2013; for a detailed review see Olsson and Martiny, 2018). Due to these recent societal changes, dominant girls might no longer be viewed as unpopular. This would suggest an influence of what Bronfenbrenner and Morris (2007) call the *meso*- and the *macrosystem* on the *microsystem* of the peer group. In order to shed more light into this matter, future studies investigating the association of prosociality and dominance could consider parental attitudes or socio-economic backgrounds of preschoolers.

Another interesting aspect of our study concerns the external validity obtained via sociometric and trait ratings made by teachers. Similarly to earlier findings by Peceguina et al. (2020), teachers' popularity ratings calculated from their assessment of friendship between the children were moderately correlated with children's sociometric interviews, thereby showing the highest agreement between any of the teacher and child measures. However, teachers' ratings about individual friendships showed extreme inconsistency, with two teachers who rated the same group sometimes agreeing perfectly, sometimes disagreeing completely. The low accuracy of teachers' friendship nominations is not entirely surprising: Studies comparing teacher and parent friendship nominations to ratings made by children themselves have found that correlations are moderate at best, with teachers and parents often identifying a lower percentage of friendships than children themselves (Altman et al., 2020; Shin et al., 2014). While teachers' dominance ratings were weakly correlated with both measures of children's dominant behavior (cf. Pellegrini et al., 2007), ratings for prosociality were not correlated with children's sharing and helping. The low accuracy of teachers' prosociality ratings could be caused by the multidimensional nature of prosociality: only one item in the questionnaire specifically asked about sharing and helping, respectively (“willingly shares with other children,” “helps other children”). The other items asked about comforting behavior and empathy, respectively. Working with a combined prosociality scale therefore might have masked connections between teacher ratings of sharing and helping with children's behavior. We propose that future studies investigating preschoolers' prosocial behavior with teacher questionnaires should not treat prosociality as a single category, but as a multidimensional construct with distinct subcategories for the different types of prosocial behavior (cf. Rodrigues et al., 2017; see also Carlo and Randall, 2002). The mixed quality of teachers' ratings in our study also adds to the notion that such ratings should be used with caution and never as a single source of information, as they are prone to being influenced by numerous factors including teachers' workload and attitude toward their environment (Pas and Bradshaw, 2014), student ethnicity (Mason et al., 2014) or personal liking of the rated child (Hughes and Chen, 2011).

Our study also had some limitations and constraints on generality (cf. Simons et al., 2017). One unexpected effect that was present in both games was that the number of children present in a group during a given session negatively influenced the number of sharing and helping acts, respectively. The larger the groups were, the less likely individual children were to share and help. Especially in the sharing game, this can likely be explained by time constraints and the availability of the toy. In a group with more children, each individual child had less time to play with and afterwards share the toy. We tried to account for the different group sizes by adjusting the duration of game 1 accordingly (i.e., 3 min for groups with <10 participants, 5 min for groups with ≥10 participants), but it is possible that this adjustment was not precise enough to counteract the effect. Since we recruited children from regular preschool classrooms that naturally differed in size, we were not able to conduct our study with groups composed of equal numbers of children. This level of control is typically more easily reached in laboratory contexts and hard to achieve in the natural environment. By including group size as a control predictor in our analyses, however, we were able to compensate for this bias. Future studies should nevertheless strive for keeping group sizes as similar as possible or find more precise ways to adjust their experimental procedures to groups of different sizes. One possible constraint on generality is that we assessed children's dominance and prosocial behavior during games. We chose games because games are a familiar part of preschoolers' everyday lives. Previous studies have successfully employed games for assessing various socio-cognitive abilities in preschool children (e.g., Theory of Mind; Priewasser et al., 2013; normativity; Schmidt et al., 2016). The games in our study were designed to be age-appropriate and to elicit prosocial and dominant behaviors in a familiar setting. Game 1 was close to naturally occurring group interactions in preschool classrooms (i.e., competing over a toy), while games 2 and 3 were similar to interactions occurring with the guidance from an adult (e.g., group play sessions with kindergarten teachers, physical education classes). Still, each game was played with a specific set of rules that do not necessarily transfer to all situations in a preschool classroom, such as free play periods. Consequently, caution must be taken when attempting to generalize our findings to children's interactions outside of the game context. Although children are known to acquire and practice social abilities via games (Rauf and Bakar, 2019), behavior shown during experimental games may not be shown in everyday preschool life, when no such rules are present. Nonetheless, results of our study have valuable educational implications, regarding activities and the general environment in preschool classrooms. Our finding that friendship favors the occurrence and, as a consequence, the development of sharing and helping, stresses the importance of friendship formation in early life. Preschool teachers should attempt to create an environment in which friendships can be formed. Teacher-instructed play could be employed to include outsiders or children new to the preschool group. Teachers could specifically instruct games that encourage friendship formation and prosocial behaviors, such as games where children need to cooperate to achieve a goal.

Overall, by adopting a developmental-ecological perspective, the current study adds to existing literature on precedents and motivations of children's prosocial behavior. Our findings showed both similarities and differences compared to previous research done in laboratory settings. This highlights the importance of studying prosocial behavior in preschoolers' natural environment, taking into account socio-ecological factors that affect children's prosocial actions and underlying decision processes. Research on children's prosocial development should attempt to find converging evidence from different contexts, such as laboratory experiments as well as observations and experiments conducted directly in children's natural environment (Horn et al., 2022). While laboratory experiments are perfectly suited for examining fine details of the proximal predictors, mechanisms, and outcomes of prosocial behavior, research in children's *microsystem* of their natural peer groups (cf. Bronfenbrenner and Morris, 2007) can give us unique insights into which social factors influence the actual use of prosocial behavior in children's everyday life. Indeed, employing more naturalistic settings and taking the social context into account has recently become the gold-standard in comparative psychology with non-human animals (Dahl, 2017; Horn et al., 2022) and its importance for developmental psychology has been stressed as well (Read and Szokolszky, 2018). Yet, it is challenging to examine children's behavior in their natural environment. Reliable measurement tools are often expensive and observation techniques are time-consuming (cf. Altman et al., 2020). Recent advances in wearable sensor technology and machine learning analytics promise an unprecedented view of complex social interactions among children in natural settings (Horn et al., 2024a), thereby enabling a deeper look at children's social relationships and interactions in their *microsystem*, the natural preschool peer group.

## Data Availability

The raw data supporting the conclusions of this article are publicly available on OSF. This data can be found here: https://osf.io/8whnj/.
